# Red Meat and Colorectal Cancer: Exploring the Potential HCA Connection

**DOI:** 10.1289/ehp.124-A189

**Published:** 2016-10-01

**Authors:** Carol Potera

**Affiliations:** Carol Potera, based in Montana, also writes for *Microbe*, *Genetic Engineering News*, and the *American Journal of Nursing*.

Epidemiological studies have found that eating red meat, especially if it is grilled or processed, is associated with increased risk for colorectal cancer.^1^ Heterocyclic amines (HCAs) and other cooking-related mutagens in meat are among several possible culprits that trigger carcinogenesis. In a new study, researchers focused on consumption of three key HCAs in the human diet, known as MeIQx, DiMeIQx, and PhIP. Although estimated HCA intake was not associated with colorectal cancer overall, people classified as having the greatest intake of PhIP from red meat (but not white meat) had a slightly increased risk of proximal colon cancers, which include tumors of the cecum and the ascending and transverse colon.^2^

**Figure:**
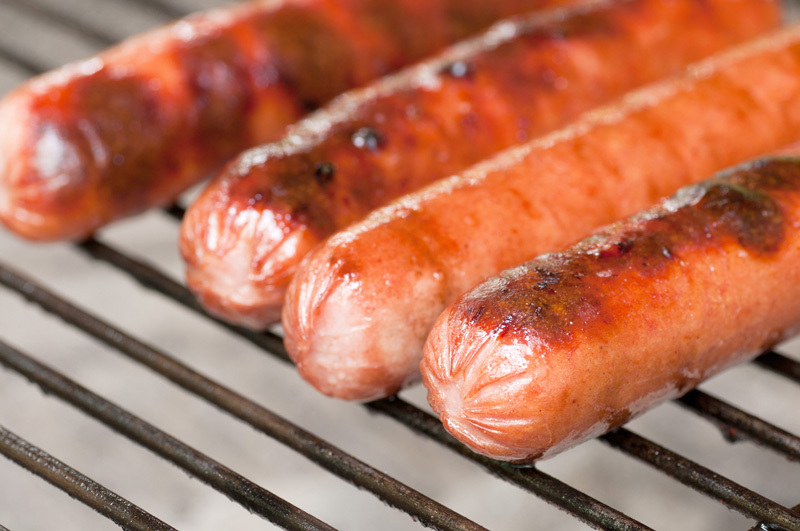
Several studies have suggested ways people might reduce their exposures to HCAs in meat: avoid cooking at very high temperatures, especially for long cooking times; use a microwave oven to partially cook meat before exposing it to high temperatures; continuously turn meat that is cooking over high heat; remove charred portions of meat before eating; and use flavorings besides meat drippings to make gravy.^9^ © Kenneth Sponsler/Shutterstock

“The association between intake of red meat, especially processed meat, and increased risk of colorectal cancer is among the most consistent findings in nutritional epidemiology,” says Lawrence Kushi, director of scientific policy at Kaiser Permanente in Oakland, California, who was not involved in the work. He says such findings are the primary basis for recommendations from the American Cancer Society^3^ and World Cancer Research Fund International^4^ to limit intake of red meat and processed meat, as well as for the classification of processed meat as a Group 1 human carcinogen by the International Agency for Research on Cancer.^5^

Despite the lack of strong positive associations, recommendations to eat less red and processed meat still make a lot of sense, says coauthor Kana Wu, a senior research scientist at the Harvard T.H. Chan School of Public Health. “At this point we cannot exclude a role of HCAs or any cooking-related meat mutagens in the development of colorectal cancer,” Wu says. Only a few prospective studies have examined the association between HCAs and colorectal cancer, with inconsistent results, and “as with every observational study, misclassification of exposure cannot be excluded,” Wu says.

The researchers used data from 29,615 men enrolled in the Health Professionals Follow-up Study and 65,785 women enrolled in the Nurses’ Health Study. Between 1996 and 2010, 418 cases of colorectal cancer occurred in men, and 790 occurred in women. The researchers asked participants about the methods they used for cooking meat, the appearance of the meat they typically ate (from “lightly browned” to “blackened/charred”), and how often they ate meat. They analyzed these data using the CHARRED (Computerized Heterocyclic Amines Resource for Research in Epidemiology of Disease) software tool developed at the National Cancer Institute. This tool enabled them to estimated participants’ intake of meat mutagens based on their meat consumption.

HCAs are just one mechanism proposed to explain the meat–cancer link. Other mechanisms, such as heme iron (the type of dietary iron found mainly in blood and meat), have not been investigated, says Wu. Red meats are rich in heme iron, which has been shown to cause preneoplastic lesions in rats.^6^ Grilling meat also produces carcinogenic polycyclic aromatic hydrocarbons, which may act in concert with HCAs in foods.^7^

Cooking-related mutagens are generated at 120–230°C, which are the temperatures needed to grill, roast, and fry meat.^8^ In contrast, cooking meat in water, such as for stews and soups, is done at temperatures of 98–120°C. “This leaves meat almost free of dietary carcinogens that result from cooking, processing, and food preparation,” says first author Ngoan Tran Le, a visiting scientist at the Harvard T.H. Chan School of Public Health. However, he adds, cooking in water “would not necessarily remove the carcinogenic potential of, for example, heme iron.”
